# Defining and tracking medical student self-monitoring using multiple-choice question item certainty

**DOI:** 10.1186/s12909-020-02250-x

**Published:** 2020-10-06

**Authors:** Mike Tweed, Gordon Purdie, Tim Wilkinson

**Affiliations:** 1 Department of Medicine, University of Otago Wellington, PO Box 7343, Wellington, 6242 New Zealand; 2 University of Otago Christchurch, Christchurch, New Zealand

**Keywords:** Self-monitoring, Certainty, MCQ assessment

## Abstract

**Background:**

Self-monitoring is an important component of clinical practice. It is underpinned by the framework of self-efficacy which is concerned with judgments of how well one believes one can achieve or perform a task. This research aimed to develop criteria for adequate self-monitoring, then to measure patterns of self-monitoring, and to explore how these patterns relate to a student’s year in a medical course and to patterns of knowledge.

**Methods:**

Analysis of individuals’ levels of correctness in answering assessment items and their certainty in correctness may be used to inform assessments of ability to self-monitor. Two criteria were proposed and applied to define adequate self-monitoring. Firstly, increasing proportions correct with increasing levels of certainty. Secondly, having a proportion correct for high certainty responses that was not lower than cohort levels. Student responses in progress tests comprising multiple-choice questions (MCQs) and associated certainty were analysed. Criteria for the presence of adequate self-monitoring and for adequate knowledge were applied to the results of each of four tests conducted over 2 years, and used to categorise patterns of self-monitoring and knowledge.

**Results:**

Data from 3 year group cohorts totalling 737 students were analysed. The majority (58%) of students demonstrated adequate knowledge and met both criteria for adequate self-monitoring across all four tests. The most advanced year group cohort had the highest rates of adequate knowledge and the highest rates of meeting both self-monitoring criteria. The patterns of self-monitoring were the same as the patterns of knowledge across the four tests for 454 students, but for the remaining 283 the patterns of self-monitoring and knowledge differed.

**Conclusion:**

Analysis of responses to item level certainty has informed development of a definition of adequate self-monitoring that may be applied to individual student’s responses from a single test, and to track the adequacy of a student’s self-monitoring over time. Patterns of self-monitoring tend to match patterns of knowledge, but not in all cases, suggesting the self-monitoring measure could provide additional information about student ability.

## Background

Self-monitoring and self-assessment underpin professional medical practice [1–4]. Self-monitoring refers to reflection-in-action of performance in the moment (e.g. do I need to check this? Am I about to do the right thing?), whilst self-assessment refers to a judgment on one’s cumulative evaluation of overall performance (e.g. do I need to refresh my understanding of the best practice for this?) [2, 3, 5].

Healthcare professional (HCP) self-monitoring is underpinned by the framework of self-efficacy [1]. Self-efficacy theory is concerned with judgments of how well one can achieve or perform in a variety of settings [6–8], as a task specific self-belief [9]. Within social cognitive theories (SCTs), self-efficacy contributes to performance by motivating people to succeed [10], thereby influencing function and behaviour [11]. Social cognitive theories have been used as frameworks to investigate and explain many human behaviours and performance including HCPs in different contexts [12]. The premise of these theories of cognitive self-regulation is that people are aware of their intellectual performance and that awareness influences their decision-making [13]. Both belief about consequences of decisions and belief about capabilities to make decisions influences behaviour [12]. These are seen as central to occupational [14], including HCP [15], practice.

HCPs require not only a considerable amount of knowledge but also accurate self-monitoring when applying that knowledge to make decisions [16]. Research into self-monitoring, and the processes underlying it, has the potential to cause a shift in educational practice, with a significant impact to improve both student learning and clinical decisions; ultimately leading to better diagnoses [17]. Given the lengthy trajectory of development as a student and then as a trainee, self-monitoring, like other important attributes, requires tracking [18].

Development of a measure of HCP’s self-monitoring would be useful, but has been under-researched [1, 2]. Specifically, despite its value, there is no definitive method to measure self-monitoring, nor to determine what is an adequate level of self-monitoring, that can be used for HCPs and/or students. Methods to assess self-monitoring have included inference from other factors in assessments, such as analysis of response times [2–4, 19]; the rates of flagging or deferring responses to questions [2–4, 19]; the rates of changing responses to questions [4]; and asking candidates to rate their certainty per item [3, 19]. However, none of these methods defined a measure of adequate self-monitoring nor tracked the adequacy of an individual’s self-monitoring over time.

We suggest that the following are required to address current gaps in the understanding of self-monitoring: (1) a working definition of adequate self-monitoring that can be derived from assessment responses; and (2) a test of concept to track changes in the adequacy of individuals’ self-monitoring over time.

This research proposes a definition of adequate self-monitoring and sets out to address three questions:

1. Could a working definition of adequate self-monitoring be captured from a single assessment?
2. How might the presence of adequate self-monitoring, as determined from this working definition, be tracked over repeated assessments?
3. How might adequate self-monitoring, as determined from this working definition, relate to concurrent measures of adequate knowledge tracked over the same repeated assessments?

## Methods

### Self-monitoring extrapolated from item level certainty responses

Considering the need to define a measure of adequate self-monitoring that can be tracked, one aspect of self-monitoring that can be used is HCPs’ awareness of their accuracy in making clinical decisions [17]. Clinical decisions are intrinsically associated with an estimate of certainty [20]. When clinicians make decisions, they need to be appropriately certain they are correct [17, 21, 22]. Certainty in excess of correctness risks error (not checking before acting on a decision), while too little certainty when one is correct can cause delays (checking every time before acting on a decision) [23].

Item level certainty has been used previously within assessment [23–26]. However, these studies used certainty based on a probability correct estimate, such as “there is a 70% probability that this answer is correct”. This absolute measure of accuracy [17] is not authentic to clinical practice [27], as clinicians do not generally consider their likelihood of the clinical decision being correct in terms of probability, but rather ask (or should ask) “Do I need to check this? Am I about to do the right thing?” [1–4]. The interaction of correctness of, with certainty in, test item responses can be extrapolated to self-monitoring when the appropriate format for certainty is used [28–33].

### Development of descriptors for item level certainty

Experienced and novice HCPs differ in their mental representations of clinical problems and decisions [34]. Training progressions in medical education involve including students and trainees in activities and decisions with increasing independence of supervision [35]. In clinical practice “specific problems are often addressed though consultations with colleagues or with medical literature” [36]. Appropriate knowledge and certainty is needed to act with increasing independence [37]. When considering self-monitoring as the degree of correctness for levels of certainty, we have used the conceptual description that frames certainty as the need to “look it up” [1, 2] or “refer this problem to another individual” [1]. We started work in this area by recording correctness for levels of certainty in multiple choice question (MCQ) tests in research settings and in lower and higher stakes assessments, and during this developed certainty rating scale descriptors [27, 38–41] that are used in this study (Table 1).

**Table 1:** Certainty descriptors for each MCQ item

No certainty	Low certainty	Moderate certainty	High certainty
I have no or insufficient experience and/or knowledge upon which to base a response. *My answer is:*effectively a guess.	I have limited experience and/or knowledge upon which to base a response. *My answer is:* based on limited information.	I have partial experience and/or knowledge upon which to base a response. *My answer is:* based on partial information.	I have sufficient experience and/or knowledge upon which to base a response. *My answer is:* based on sufficient information.
I would need to consult a colleague, clinician, or references prior to considering any response.	I would need to consult a colleague, clinician or references for assistance in formulating my response.	I would need to consult a colleague, clinician or references to confirm the appropriateness of my response.	I would have no need to consult a colleague, clinician or reference in order to make a response.
In an authentic healthcare situation, I would **require education to respond**.	In an authentic healthcare situation, I would **require direction to respond**.	In an authentic healthcare situation, I would **require confirmation to respond**.	In an authentic healthcare situation, I would be **able to respond**.
			While I may consult a colleague or clinician, this is because they are required to undertake further action, not to educate, direct, or confirm my response.

Certainty descriptors were presented to the students within assessment documentation for the year and at the start of each test

### Adequate self-monitoring defined from responses including item level certainty

A definition for adequate self-monitoring was developed from two initial criteria.

The rationale for the first criterion built on the assumption that an individual’s self-monitoring would be demonstrated if their odds of responses to test items being correct increased as their levels of certainty in those responses increased [27, 40], where questions answered with higher certainty were more likely to be answered correctly than questions answered with lower certainty [40]. Our first criterion was therefore defined as a statistically significant trend for increasing proportions correct with increasing levels of certainty.

For the second criterion, we worked on the assumption that for questions answered with high certainty we would expect to see a high proportion answered correctly, particularly as the descriptor for high certainty included taking action without any need for checking with colleagues or a reference (Table 1). Having a criterion of the proportion correct for high certainty responses being statistically significantly higher than a given level would be difficult for those with a low number of high certainty responses to achieve. We decided a lack of self-monitoring would be indicated by an individual student’s correctness for high certainty responses being statistically significantly lower than the cohort’s overall proportion correct for high certainty responses. The second criterion therefore, was not having a statistically significantly lower proportion of correct answers for high certainty responses than the overall cohort.

From the arguments and assumptions outlined above, we propose that an individual student on an individual test would be classified as having adequate self-monitoring if the following criteria were met:

1. A statistically significant trend for increasing proportions correct with increasing levels of certainty

AND

2. Not having a statistically significant lower proportion correct for high certainty responses than the overall cohort.

### Test of concept

For the purposes of this study, we used student responses to computer-delivered multiple choice question (MCQ) progress tests to investigate, as a test of concept, this working definition of self-monitoring.

### Context

The MB ChB degree at the University of Otago is a 6-year course. Year 1 is a common health science course. The curriculum in Years 2 and 3 includes a mixture of small group, lecture, self-directed, and simulated clinical skills learning experiences, with minimal authentic clinical contact. Years 4 and 5 are completed at one of three geographically separate campuses, and include learning in clinical environments complemented by a mixture of small group, lecture, and self-directed learning experiences. Year 6 is a Trainee Intern year, with students placed as members of healthcare teams undertaking a variety of duties under supervision across many different health care locations.

The MCQ progress test is computer-delivered. All students sit the test twice each year, in April and September, in Years 2–5. Each MCQ progress test consists of 150 items purposefully selected to cover a range of content from a pool of 700 items related to the core curriculum. These 150 item tests are delivered in random order to each student, each with 5–16 possible response options, including a single most correct answer. Following each question response, the students complete a certainty rating based on descriptors of no, low, moderate or high certainty (Table 1). The certainty rating descriptors (Table 1) are presented to students at the beginning of each year and again at the beginning of each test.

The test is not administered under examination conditions: students have a 2-week window to complete the test in their own time. The number of correct answers on these MCQ progress tests is assumed to be an indicator of student knowledge. There is no formula scoring. The minimum satisfactory knowledge standards for each year group for each test are calculated using Taylor’s modification of the Cohen method [42]: specifically, this is 0.65 x total correct by students at 90th centile for that year group for each test.

Students receive feedback on their performance in each test 2 weeks after the test closes. The minimum satisfactory standards for numbers correct for each year group are provided. Individual feedback includes their overall proportion correct, proportions correct for each level of certainty, and proportions correct by curriculum subjects. The students do not receive item level feedback.

The primary purposes of the progress test include giving students an indication of their current performance in relation to the minimum satisfactory standard for their cohort, and of how their performance changes as they progress through the course. There is no impact on summative decisions, provided the students demonstrate engagement in the progress test as an educational activity.

### Student participants

Data for this study were derived from tests sat by 3 cohorts of students who were in Years 2 to 4 and subsequently in Years 3 to 5. Students were excluded if they repeated or missed a year, or did not sit all four tests within the 2-week window.

### Patterns of meeting self-monitoring criteria and knowledge across four tests

Each student’s certainty in and correctness of responses allowed for determination of whether self-monitoring criteria were met on each occasion of the four tests. These were categorised into one of five patterns:

4. Consistent self-monitoring: the student met the self-monitoring criteria in each of the four tests.
5. Improving self-monitoring: one or both criteria were initially not met, and at any subsequent test both self-monitoring criteria were met and were also met for all subsequent tests,
6. Not self-monitoring: the student did not meet the self-monitoring criteria in any of the four tests.
7. Declining self-monitoring: having initially been met, self-monitoring criteria were then not met, and were also not met for all subsequent tests.
8. Inconsistent self-monitoring: any remaining pattern.

Each student’s total correct responses allowed for determination of whether knowledge criteria (score above the minimum satisfactory standard for that time in the relevant year) were met on each occasion of the four tests. These were categorised into one of five patterns:

1. Consistent knowledge: score met the relevant standard for all four tests
2. Improving knowledge: score initially less than relevant standard, and at any subsequent test met the standard and also met the relevant standard for all subsequent tests
3. Low knowledge: scores below the relevant standard on all four tests
4. Declining knowledge: initially score(s) met relevant standard(s), then were below the relevant standard and remaining so for all subsequent tests
5. Inconsistent knowledge: any remaining pattern

Therefore, each individual student had a pattern of self-monitoring and a pattern of knowledge across the four tests.

### Statistical analysis

Firstly, whether a student was adequately self-monitoring for each of the tests was determined by whether both criterion 1 and criterion 2 for adequate self-monitoring were met. For each test, for each student, a two-tailed exact Cochran-Armitage test for trend was used to test for criterion 1, testing for significantly increasing proportions correct with increasing certainty. A two-tailed exact binomial test was used to determine whether the proportion correct for high certainty responses in each student’s test was significantly different to the proportion for the cohorts combined. If the test was not significant, or if significant the proportion correct for that test, for that student, was higher than the proportion correct for the cohorts combined then criterion 2 was met.

Subsequent statistical analysis focussed on students’ self-monitoring and knowledge patterns across the four tests.

A chi-squared test was used to compare the distribution of self-monitoring patterns between class groups. The proportions with improving and declining self-monitoring patterns were compared with a binomial test.

The Fisher’s exact test extension, the Freeman-Halton exact test, was used to compare the proportions in a contingency table of the patterns of adequate self-monitoring and knowledge. The *p*-value was estimated with Monte Carlo estimation with 50,000 replications.

When a test for a contingency table was significant, significant cells were identified using standardised residuals, adjusting for multiple comparisons with the Holm–Bonferroni method.

SAS 9.4 (SAS Institute Inc., Cary, NC, USA) was used for the analysis. A *p*-value < 0.05 was considered statistically significant.

## Results

### Broad descriptions

Of the 899 students in the 3 cohorts, 162 were excluded as they had not completed all four tests within the timeframe for each test.

Of the 737 students included in the study, 252 were in the year 2–3 cohort, 237 in year 3–4 and 248 in year 4–5. The levels of certainty, correctness, being above the minimum standard for knowledge are shown in Table 2. Overall 84.2% of high certainty responses were correct.

**Table 2:** MCQ Items correctness and certainty responses, students meeting knowledge standard and self-monitoring

	Class Y2–Y3 2015 *N* = 74,628 2016 N = 74,652	Class Y3–Y4 2015 *N* = 70,000 2016 N = 70,463	Class Y4–Y5 2015 *N* = 73,548 2016 N = 73,582
Certainty 2015			
No	36.4% (27200)	39.7% (27818)	17.8% (13077)
Low	40.8% (30419)	21.2% (14830)	37.6% (27660)
Moderate	14.2% (10600)	13.5% (9437)	26.6% (19530)
High	8.6% (6409)		18.1% (13281)
Certainty 2016			
No	34.7% (25939)	27.4% (19293)	14.1% (10342)
Low	35.2% (26258)	34.3% (24142)	34.3% (25218)
Moderate	17.1% (12787)	22.1% (15604)	27.8% (20483)
High	13.0% (9668)	16.2% (11424)	23.8% (17539)
Correct answers 2015	37.5% (27998)	46.2% (32321)	55.5% (40826)
By certainty			
No	27.0% (7345)	33.4% (5984)	37.1% (4853)
Low	30.8% (9382)	35.2% (9779)	42.4% (11720)
Moderate	58.7% (6227)	60.0% (8899)	65.6% (12814)
High	78.7% (5044)	81.2% (7659)	86.1% (11439)
Correct answers 2016	42.2% (31468)	51.8% (36501)	61.1% (44985)
By certainty			
No	28.4% (7354)	39.1% (7534)	40.1% (4150)
Low	33.1% (8688)	40.2% (9695)	45.0% (11341)
Moderate	58.6% (7497)	62.3% (9724)	68.7% (14063)
High	82.0% (7929)	83.6% (9548)	88.0% (15431)
Above the standard	N = 252	N = 237	N = 248
2015 May	78.2% (197)	92.0% (218)	94.8% (235)
2015 September	75.8% (191)	86.9% (206)	95.2% (236)
2016 May	71.0% (179)	91.6% (217)	96.0% (238)
2016 September	83.3% (210)	91.1% (216)	97.6% (242)
Self-monitoring	N = 252	N = 237	N = 248
2015 May	87.7% (221)	82.3% (195)	93.1% (231)
2015 September	83.7% (211)	85.2% (202)	91.5% (227)
2016 May	86.1% (217)	81.0% (192)	88.7% (220)
2016 September	86.1% (217)	85.7% (203)	95.6% (237)

N for certainty and correct are the total number of questions answered by all students in both tests. N for above the knowledge standard and self-monitoring are the number of students

In addition, adequacy of meeting the self-monitoring criteria on individual tests are also shown for descriptive purposes, by cohort and calendar year in Table 2. Across all 2948 individual test results, 87.3% demonstrated adequate self-monitoring. Criterion 1 but not criterion 2 was met in another 5.7% of test results. These 5.7% of test results indicate a significant trend for increasing proportions correct with increasing levels of certainty, but a significantly lower proportion correct for high certainty responses than the cohorts combined.

### How does adequacy of self-monitoring change over repeated tests

In addressing research question 2, the self-monitoring pattern of the majority of the students (73.7%; 95% confidence interval (CI) 69.9–76.4%) did not change over the 2 years of the study: 70.8% (522/737) consistently self-monitored and 2.4% (18/737) were consistently not self-monitoring. There was no significant difference between the proportion of students with an improving self-monitoring pattern (7.2%; 95%CI 5.4–9.3%) and the proportion with a declining self-monitoring pattern (5.6%; 95%CI 4.0–7.5%; *p* = 0.26, binomial test) (Table 3).

**Table 3:** Prevalence of patterns of self-monitoring by class cohort groups

Self-monitoring pattern	Class Y2–Y3 ***N*** = 252	Class Y3–Y4 ***N*** = 237	Class Y4–Y5 ***N*** = 248	Total ***N*** = 737
**Consistent self-monitoring**	70.2% (177)	63.3% (150)	78.6% (195)	70.8% (522)
**Improving self-monitoring**	6.7% (17)	10.5% (25)	4.4% (11)	7.2% (53)
**Not self-monitoring**	2.8% (7)	3.4% (8)	1.2% (3)	2.4% (18)
**Declining self-monitoring**	7.5% (19)	6.3% (15)	2.8% (7)	5.6% (41)
**Inconsistent self-monitoring**	12.7% (32)	16.5% (39)	12.9% (32)	14.0% (103)

Significant differences in self-monitoring were found between cohort groups (χ^2^ = 19.9, df = 8, *p* = 0.011) (Table 3). Students who had been in the course for 3–4 years had significantly lower rates of consistent self-monitoring than the cohort of students who had been in the course for 4–5 years. No significant differences were found between the year 2–3 and year 3–4 cohorts or between the year 2–3 and year 4–5 cohorts.

### Relationships between knowledge and self-monitoring

In addressing research question 3, Table 4 shows the relationships between knowledge and self-monitoring, most of which were equivalent patterns. The distributions of patterns of meeting the knowledge standard and patterns of self-monitoring were not independent (*p* < 0.0001, Freeman-Halton test). Those students with patterns of consistent self-monitoring were significantly more likely also to be consistently above the minimum satisfactory knowledge standard, and significantly less likely to have any other knowledge pattern. Similarly, those consistently above the minimum satisfactory knowledge standard were significantly less likely to have any self-monitoring pattern other than consistent. As expected, significant relationships were found between improving self-monitoring and improving knowledge; declining knowledge and declining self-monitoring; low knowledge and not self-monitoring; low knowledge and declining self-monitoring; and between inconsistent knowledge and inconsistent self-monitoring.

**Table 4:** Relationship of meeting knowledge standard patterns and self-monitoring patterns

	Consistent self-monitoring	Improving self-monitoring	Not self-monitoring	Declining self-monitoring	Inconsistent self-monitoring	Total
**Consistent knowledge**	428	26	4	21	53	532
	82.0%	49.1%	22.2%	51.2%	51.5%	72.2%
	80.5%	4.9%	0.8%	3.9%	10.0%	100%
**Improving knowledge**	17	15	3	1	10	46
	3.3%	28.3%	16.7%	2.4%	9.7%	6.2%
	37.0%	32.6%	6.5%	2.2%	21.7%	100%
**Low knowledge**	1	2	4	4	4	15
	0.2%	3.8%	22.2%	9.8%	3.9%	2.0%
	6.7%	13.3%	26.7%	26.7%	26.7%	100%
**Declining knowledge**	12	2	2	7	9	32
	2.3%	3.8%	11.1%	17.1%	8.7%	4.3%
	37.5%	6.3%	6.3%	21.9%	28.1%	100%
**Inconsistent knowledge**	64	8	5	8	27	112
	12.3%	15.1%	27.8%	19.5%	26.2%	15.2%
	57.1%	7.1%	4.5%	7.1%	24.1%	100%
**Total**	522	53	18	41	103	737
	100%	100%	100%	100%	100%	
	70.8%	7.2%	2.4%	5.6%	14.0%	

First row: Number of students

Second row: Percentage of students in self-monitoring group

Third row: Percentage of students in knowledge group

The distributions of patterns of meeting the knowledge standard and patterns of self-monitoring were not independent (p < 0.0001, Freeman-Halton test)

Of the 737 students, 454 had patterns of self-monitoring that were the same as the patterns of knowledge (consistent knowledge and self-monitoring patterns, improving knowledge and self-monitoring, below the standard and not self-monitoring and declining knowledge and self-monitoring). The remaining 283 had a pattern of self-monitoring that was different to the pattern of knowledge. Of these 283 students where patterns were not the same, 188 had an inconsistent pattern of either knowledge or self-monitoring (Table 4).

## Discussion

A two-criteria working definition of adequate self-monitoring has been proposed related to self-monitoring in individual test results. Subsequently, as a test of concept, this has been used to track self-monitoring for individual medical students across four tests. For 87.3% of tests, individual students were classified as self-monitoring. Most (58%; 428/737) of the students met both the expected minimum knowledge standard on all four tests and met both self-monitoring criteria across all four tests. The Year 4–5 cohort of students, with the greater experience and knowledge, had the highest rates of meeting both self-monitoring criteria. Though the findings imply that knowledge and self-monitoring patterns generally align, there are still substantial numbers of students with different patterns. These different patterns could be due to random variation, when the patterns should align, or that the knowledge and self-monitoring criteria are addressing different constructs. A measure of adequacy of self-monitoring that can be tracked over time, would be a useful addition to information on adequacy of knowledge (derived from numbers correct) in assessing students.

We have no gold-standard of adequacy of self-monitoring with which to compare the criteria we used, but suggest we have started to provide some evidence. The proposed two-criteria working definition of adequate self-monitoring (derived from correctness for levels of certainty stratified by the need to consult a colleague or reference) is a coherent fit with the concept of self-monitoring [1–4]. The argument for measures of knowledge and measures of self-monitoring addressing different constructs is based on authenticity to clinical practice [1, 2], underpinned by self-efficacy theory [6–8]. This research uses an item level certainty scale anchored with descriptors of the need to seek support for a decision by checking with a resource or colleague, thereby aligning to self-monitoring judgment [1, 2], authentic to clinical practice [15, 20]. This self-monitoring judgment is a self-efficacy judgment and a task specific self-belief [9] which is needed to achieve or perform effectively [6–8]. By having the certainty decision as a second decision, after the option choice, the students are making the certainty decision based on that option choice. Therefore, this decision-making ensures incorporation of their awareness of their intellectual performance [13], and their belief about their capabilities [12].

The first criterion is based on correctness increasing as levels of certainty increase [27, 33, 38–41]. An alternative analysis could have been increasing certainty with increasing levels of correctness [28–32]. Although both these analyses could be valid, our favoured one is better aligned to self-monitoring for safe decision making in practice whereas the latter is better aligned to efficient decision making [43].

The second criterion compared the individual student’s proportion correct for high certainty responses with that of the combined cohort. This does introduce a degree of peer-referencing within the definition of adequate self-monitoring, however the likelihood of being correct for answers given with high certainty has consistently been in the 80–90% range over several student groups, with different year groups and cohorts [27, 39–41] and was 84% in this study.

One additional criterion for adequacy of self-monitoring might be the odds of unsafe responses amongst the high certainty responses [27], as these would result in errors with the greatest impact in clinical practice. Adding the potential safety of responses to item level certainty [30–32] has been included in other research programmes. Another criterion to consider might relate to too high a proportion correct for no certainty [43], as this may indicate inefficiency in self-monitoring. It would be possible to generate an additional metric based on certainty when correct [31, 32, 44], but the analysis we describe prioritises safety (being correct when certain) over efficiency (being certain when correct) [43].

Potential limitations to this study include low numbers of students in some combinations of self-monitoring and knowledge patterns. If there are any relationships involving these combinations, this study lacks the power to find them. There were a number of students whose patterns for both self-monitoring and/or knowledge were categorised as inconsistent: this could be an accurate assessment or indicate that the current assessments were not sufficiently robust to detect their true self-monitoring and knowledge patterns. Likewise, the finding of no evidence of a difference in the numbers of students whose self-monitoring patterns improved or declined over the four tests might be due to insufficient numbers of students with these response patterns.

A further limitation is that the decisions being self-monitored were made on a test delivered within a 2 week time frame and were therefore less authentic to in-the-moment decisions related to patients. However we need to introduce and track this for HCP students, without the potential risk to patient safety that authentic clinical decision making would bring.

To support these criteria for assessing adequacy in self-monitoring and tracking patterns of self-monitoring over time, we suggest additional investigations that would add validation evidence. These would include exploring associations with other measures used to infer self-monitoring, investigating in other test formats, and application to more than 2 years of a course. Another area would be to explore the educational impact of item level certainty feedback and the development and maintenance of self-monitoring [17, 33].

### Practice points

1. We have proposed a definition of adequate self-monitoring using two criteria based on responses from MCQs using item level certainty. This definition for adequate self-monitoring can be applied to individual students on individual tests and tracked across several tests.
2. Those who have a pattern of adequate self-monitoring also tended to have a related pattern of adequate knowledge and vice versa. However, there were exceptions, with several combinations of patterns of self-monitoring and knowledge. This may suggest these measures are different and provide additional information.
3. We suggest that this measure of adequacy of self-monitoring can be tracked over time is a useful addition to information in assessing students.

## Data Availability

The datasets generated and/or analysed during the current study are not publicly available due to the stipulations the ethics approval.

## Abbreviations

CI: Confidence Intervals
HCP: Healthcare Professional
MCQ: Multiple Choice Question
